# Meta-analysis of the efficacy of digital therapies in children with attention-deficit hyperactivity disorder

**DOI:** 10.3389/fpsyt.2023.1054831

**Published:** 2023-05-16

**Authors:** Fan He, Yanjie Qi, Yuanyue Zhou, Aihua Cao, Xin Yue, Shuanfeng Fang, Yi Zheng

**Affiliations:** ^1^Beijing Key Laboratory of Mental Disorders, Beijing Anding Hospital, Capital Medical University, Beijing, China; ^2^Department of Medical Psychology, The First Affiliated Hospital, Hainan Medical University, Haikou, Hainan, China; ^3^Qilu Hospital, Shandong University, Jinan, Shandong, China; ^4^MaiDeHaiKe Technology, Beijing, China; ^5^Children's Hospital Affiliated, Zhengzhou University, Zhengzhou, Henan, China

**Keywords:** digital therapy, attention-deficit hyperactivity disorder, inattention, impulsive hyperactivity, executive function, working memory, meta-analysis

## Abstract

**Background:**

Attention deficit hyperactivity disorder (ADHD) is a neurodevelopmental disorder that commonly occurs in childhood. The aim of this meta-analysis was to summarize the available evidence for the efficacy of digital therapeutics in children and adolescents with ADHD.

**Methods:**

We searched the MEDLINE, EMBASE, Cochrane Library (Cochrane Database of Systematic Reviews), and Web of Science (science and social science citation index) databases for relevant studies and used Stata 15.0 software to carry out the meta-analysis.

**Results:**

A total of 31 studies involving 2169 participants (1665 boys and 504 girls) aged 4–17 years old were included in the final analysis. The meta-analysis results showed that digital interventions improved the symptoms of inattention with an effect value of −0.20 (95% confidence interval [CI] −0.36, −0.04) and decreased the continuous performance task (CPT) reaction time (effect, −0.40, 95% CI −0.73, −0.07) in ADHD patients. The score for impulsive hyperactivity was slightly decreased (effect, −0.07, 95% CI −0.23, 0.09). Moreover, executive function was improved (effect, 0.71, 95% CI 0.37, 1.04). The capability of working memory appeared to be increased (effect, 0.48, 95% CI 0.21, 0.76) between the two groups. Visual appraisal of the sensitivity analysis suggested the absence of heterogeneity, and no obvious publication bias was detected.

**Discussion:**

Based on the existing literature evidence, we conclude that digital therapy can be a promising therapeutic strategy for ADHD patients.

## Introduction

Attention deficit hyperactivity disorder (ADHD) is a neurodevelopmental disorder that commonly occurs in childhood and is characterized by inattention, hyperactivity, and impulsivity. The prevalence of ADHD in children and adolescents ranges from 4.2–6.5%, and in 30–50% of cases, the symptoms persist into adulthood (1). To date, the etiology and pathogenesis of ADHD are incompletely understood, although genetic, environmental, brain developmental and psychosocial factors have been identified. The behavioral symptoms, cognitive dysfunction, and comorbidity of ADHD pose many problems for the ability of affected children to learn and carry out activities of daily life, causing a heavy burden on families and society. Therefore, systematic and standardized therapies are needed for ADHD.

At present, the main treatments for ADHD are drug therapies. Methylphenidate, dexamphetamine and atomoxetine are the drugs most often prescribed to treat ADHD in children and adolescents. However, the most common side effects were decreased appetite with possible weight loss, irritability, palpitations, and headache. More importantly, the medications are typically used in an attempt to ameliorate the behavioral symptoms but are not designed to address skill deficits and cognitive functions (2, 3). In addition, drug resistance, possible risk of addiction, low compliance, side effects, and adverse effects are concerns of patients and their parents, all of which support the urgent need for effective alternative therapies (4, 5).

Fortunately, non-pharmacological strategies have been introduced and widely applied in the field of ADHD interventions, ranging from behavior intervention, physical therapy, neurofeedback to counseling, and more recently digital therapy. The current evidence on non-pharmacological treatments for ADHD indicates that these interventions can lead to improvements in self-reported ADHD symptoms as well as in the symptoms reported by the parents and teachers of children (6, 7). The combination of medication management and behavioral therapy leads to significantly greater satisfication with treatment plans and allows for the use of lower stimulant dosages, possibly reducing the risk of adverse effects, which makes such therapies more easily accepted by patients and their parents (6, 7).

Non-pharmacological therapy is designed to be administered in a long-term setting to help ADHD patients acquire psychosocial skills or improve cognitive functioning by boosting their motivation, organization/planning skill, and adaptive thinking. A new and emerging form of non-pharmacological therapy known as prescription digital therapy is in the preliminary phase of development. According to the definition provided by the Digital Therapeutics Alliance, digital therapeutics (DTx) “deliver medical interventions directly to patients using evidence-based software therapeutic interventions to treat, manage, and prevent a broad spectrum of diseases and disorders” (8, 9). The interventions can be a standalone software program or a program used in combination with self-help therapies such as exercise therapy or dietary therapy, or with hardware-assisted therapies including neurofeedback training. For ADHD treatment, different digital therapeutic strategies have been designed to improve impairment in cognitive functions or attention control found in ADHD. In 2020, the U.S. Food and Drug Administration (FDA) formally approved EndeavorRx (AKL-T01), the first video game delivered through a video game-like interface for at-home play for the treatment of ADHD in children aged 8–12 years. In a proof-of-concept study, attention and memory performance were improved significantly in patients with ADHD who received this therapy, and minimal adverse events occurred (10).

Recent growing evidence suggests that digital interventions offer effective strategies for resolving the psychological problems and for improving the attentional and working memory performance of children and adolescents with ADHD, while posing minimal risk for adverse events (11). Based on the psychological characteristics of pediatric patients, digital therapy combined attention training techniques and neurobehavioral therapy for developmental disorders and is more adaptable for ADHD children for their improvement of psychosocial skills and neurocognitive functions. Moreover, when digital therapy is combined with medication, the combined treatment was found to lead to greater improvements of academic and conduct measures, compared to medication alone, in ADHD patients with comorbid anxiety and who live in a lower socioeconomic environment (11).

However, due to the variety of digital interventions and limited sample sizes, the published studies regarding the efficacy of digital therapeutics have provided inconsistent findings (12). Therefore, it is necessary to conduct a pooled analysis of the efficacy of digital therapeutics based on a comprehensive literature search to reach a more objective conclusion. In the present study, in order to comprehensively evaluate the clinical effects of digital therapy, we define digital therapy from the broad sense as a spectrum of therapeutic measures supported by various forms of digital technology, including big data, artificial intelligence, sensor technology, video-game and virtual reality on different platforms and we aimed to review recent developments in digital therapy and to categorize different types of digital interventions. We performed a multi-dimensional evaluation of the clinical efficacy of digital therapy for symptomatic and functional improvements in ADHD patients. Finally, we compared the clinical efficacy of different digital interventions in different patient groups via subgroup analysis.

## Methods

### Literature search

In this meta-analysis, the Preferred Reporting Items of the Systematic Reviews and Meta-analysis (PRISMA) guidelines and the Cochrane Handbook of Systematic Reviews of Interventions were followed (13, 14). We searched the following English language electronic bibliographic databases: MEDLINE, EMBASE, The Cochrane Library (Cochrane Database of Systematic Reviews), Psych info and Web of Science (science and social science citation index) for relevant studies published through July 2022. The aim of this study was to include as many studies as possible on the use of digital therapy to treat ADHD patients to compare the clinical effects between digital therapy and other interventions on different clinicopathological aspects of ADHD. The search terms used included: “attention-deficit hyperactivity disorder,” “ADHD”, “information technology”, “digital”, “remote”, “multimedia”, “serious games”, “artificial intelligence”, “algorithm”, “mobile devices”, “computer program” or “computer training”, “human computer interaction”, “programmed instruction”, “communication aid”, “nonpharmacological”, “adaptive training” or “digital assistant”, “digital therapy”, “telemedicine”, “cognitive training”, “cognitive remediation”, “executive function training”, “pediatric” or “school-age children” or “school children” or “adolescent” in full or truncated versions. This project was registered in PROSPERO (https://www.crd.york.ac.uk/PROSPERO) with registration number CRD42022350349.

### Inclusion criteria

For the meta-analysis, we searched all quantitative and qualitative studies, reports, letters, reviews, editorial articles, or conference abstracts examining the efficacy of digital therapeutics in pediatric ADHD. The following inclusion criteria were applied: (1) clinical trials or prospective studies; (2) pediatric participants aged 4–17 years with a clear primary diagnosis of ADHD; (3) digital intervention that is directly provided to ADHD patients online or through mobile application via computer, phone, or tablet, to treat or manage ADHD. The intervention can be a standalone software program or a program used in combination with self-help therapies or with hardware-assisted therapies; and (4) outcomes reported as measurable continuous variables. Studies and patient populations were excluded for the following reasons: (1) comorbidity of neurodevelopmental disorder with ADHD; (2) studies that have overlapping or duplicate samples; and (3) studies with poor designs or data unavailable for extraction.

### Data extraction and quality assessment of included studies

For the meta-analysis, two reviewers independently evaluated the final included papers using a standardized form. Data extraction from each study was conducted separately by two reviewers, who then double-checked the results. The authors of the original studies were contacted with questions to identify the study's unique execution procedure, if necessary. If disagreement occurred, it was resolved through conversation or with the help of a third investigator. The following data were collected: first author's name, year of publication, country/region, age, gender ratio, years of follow-up, and research sample size. The primary outcomes of this study were the effectiveness of digital therapeutic interventions for the core symptoms (inattention, hyperactivity/impulsivity) of ADHD as determined by assessment tools including the ADHD-Rating Scale (ADHD-RS). The secondary outcomes were attention control evaluated by Continuous Performance Task (CPT) tests and executive functions and working memory assessed by the Behavior Rating Inventory of Executive Function questionnaire (BRIEF). The Newcastle-Ottawa Quality Assessment Scale (NOS) was selected to assess the methodological quality of included studies. Three major components of each study were examined: patient selection, the comparability of the intervention and the observation groups and outcome assessment. NOS score ranges from 0 to 9, with high quality defined as a NOS score ≥7.

The ADHD-RS is based on the Diagnostic and Statistical Manual of Mental Disorders, Fourth Edition (DSM-IV) criteria for ADHD and consists of inattentive items, hyperactivity/impulsive behavioral items for parents and teachers graded on a scale. For this study, we used the subscales inattention and hyperactivity as dependent variables, and other subscales or scales not rated by parents/teacher were not considered.

The CPT is a series of computerized tests of attention and hyperactivity/impulsive behavior and several versions including the Conners CPT (CPT-II, CPT-III), Integrated Visual and Auditory Continuous Perform Test (IVA-CPT), and Test of Variables of Attention (TOVA) have been developed since the 1950s. Even when different CPT versions were used, the tests typically measure reaction time, number correct, and errors of omission and commission, which correlate with inattention and impulsivity, and thus, pooled analysis could be performed. For this study, due to the limitation of the included sample size, we used the reaction times as dependent variables.

The Behavior Rating Inventory of Executive Function (BRIEF) is a rating scale for parents and teachers to assess eight sub-domains: Inhibit, Shift, Emotional Control, Initiate, Working Memory, Plan/Organize, Organization of Materials, and Monitor with 75 items. For this study, we used the BRIEF2 subscale Working Memory and the Total Scale, which are associated with ADHD severity as dependent variables, and other forms of scales were not considered.

### Statistical analysis

The meta-analysis was performed using Stata 15.0. ADHD patients who completed digital interventions were compared with controls who received no intervention or another type of intervention. Differences in ADHD-RS and BRIEF scores were analyzed for significant. CPT reaction times from different platforms were normalized according to the mean values from different studies and were applied for pooled analysis. Different meta-analyses were performed with stratification by the type of intervention (digital intervention or without digital intervention). Effects between digital intervention and control groups, and their 95% confidence intervals (CIs) were calculated using a continuous random effects model. The I^2^ test and Cochran's Q-statistic were used to assess heterogeneity among the included studies based on the odds ratio (OR) and 95% CI. Funnel plots were created, and publication bias was assessed using Begg and Egger regression. Statistical significance was defined by a *P* value < 0.05.

## Results

### Identification of relevant studies

**Figure 1:**
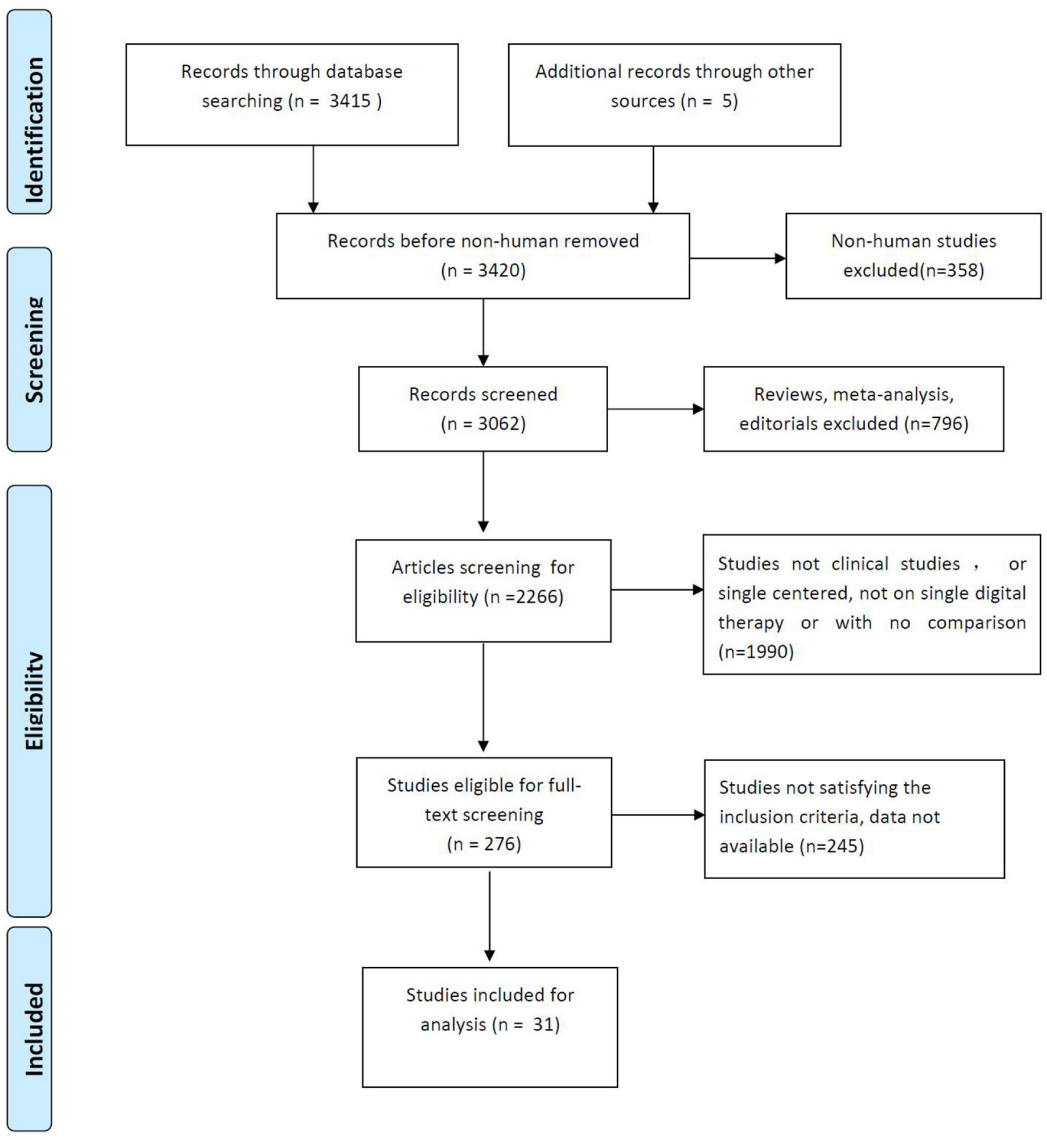
Screening process for study inclusion.

We identified 31 English language publications focusing on the efficacy of digital therapeutics in pediatric ADHD. The results of the selection process from our literature search are presented in Figure 1. The initial search returned a total of 3,420 studies, but ultimately only 31 studies met the inclusion criteria. These studies, with 2,189 participants (1,681 boys and 508 girls), were included in the meta-analysis (10, 15–43), and the characteristics of the included studies are presented in Table 1.

**Table 1:** Characteristics of the studied included in the meta-analysis.

**References**	**Region**	**Digital interventions**	**Age (Mean)**	**Sex (%Male)**	**Digital Intervention Sample**	**Control Sample**	**NOS**
Shalev et al. (44)	UK	CPAT program	9.1	83.3	20	16	7
Gevensleben et al. (43)	Germany	NF training	9.6	78.6	38	23	7
Beck et al. (45)	USA	WM training	11.7	69.2	27	24	7
Prins et al. (42)	Netherlands	RoboMemo	9.46	82.3	27	24	7
Steiner et al. (46)	USA	NF training	8.4	67.6	13	15	7
Lim et al. (39)	Singapore	BCI system	8.7	65.2	17	NA.	8
Bioulac et al. (38)	France	Secret Agent, Bubble Pop, and Kung 2	9.6	78.6	26	16	8
Chacko et al. (37)	UK	CWMT active	8.4	77	44	41	9
van der Oord et al. (35)	Netherlands	Braingame brian	9.7	66.7	18	22	7
Steiner et al. (47)	USA	NF training	12.4	52.2	31	36	7
Dovis et al. (34)	Netherlands	Braingame Brian	9.6	78.6	31	30	7
Bul et al. (33)	Netherlands	Plan-It Commander	9.85	80.6	88	82	8
Kermani et al. (32)	Iran	Continued Placement game	9.85	78.6	30	30	7
Weerdmeester et al. (31)	Netherlands	Adventurous Dreaming High?ying Dragon	9.76	79.4	34	36	7
Benzing et al. (25)	Switzerland	Xbox Kinect	9.6	78.6	28	23	7
Johnstone et al. (48)	Australia	Focus Pocus	9.6	78.6	44	41	7
Bikic et al. (29)	Denmark	ACTIVATE™	9.9	84.5	35	35	9
Bul et al. (28)	Netherlands	Plan-It Commander	9.9	82	79	64	9
Qian et al. (27)	Singapore	BCI-based attention training game system	9.6	100	11	18	7
Weisman et al. (26)	Israel	Icon™ Mobile APP	9.56	69.2	19	20	7
Ackermann et al. (49)	Switzerland	CWMT program	13.7	73.7	18	10	7
García-Baos et al. (24)	Spain	RECOGNeyes	11.05	64.3	14	14	8
Hahn-Markowitz et al. (22)	Israel	Cog-Fun	8.5	71	50	49	8
Kollins et al. (21)	USA	AKL-T01	10.6	74.3	164	173	9
Rajabi et al. (20)	Iran	NF training	10.1	100	16	16	8
Meyer et al. (50)	USA	SST training	9.6	72	20	20	7
Kollins et al. (11)	USA	AKL-T01	10.5	74.1	130	76	9
Medina et al. (10)	Spain	KAD_SCL_01	9.6	78.6	14	14	7
Ha et al. (18)	South Korea	DoBrain app	6.69	66.7	12	9	8
Luo et al. (17)	China	Focus Pocus	9	87.3	27	28	7
Mozaffari et al. (16)	Iran	RehaCom	9.6	100	20	20	7

CPAT, computerized progressive attentional training; CWMT, Cogmed Working Memory Training; WM, working memory; NF, neurofeedback; BCI, brain-computer-interface.

### Inattention improvement based on ADHD-RS score after treatment with digital therapeutics

**Figure 2:**
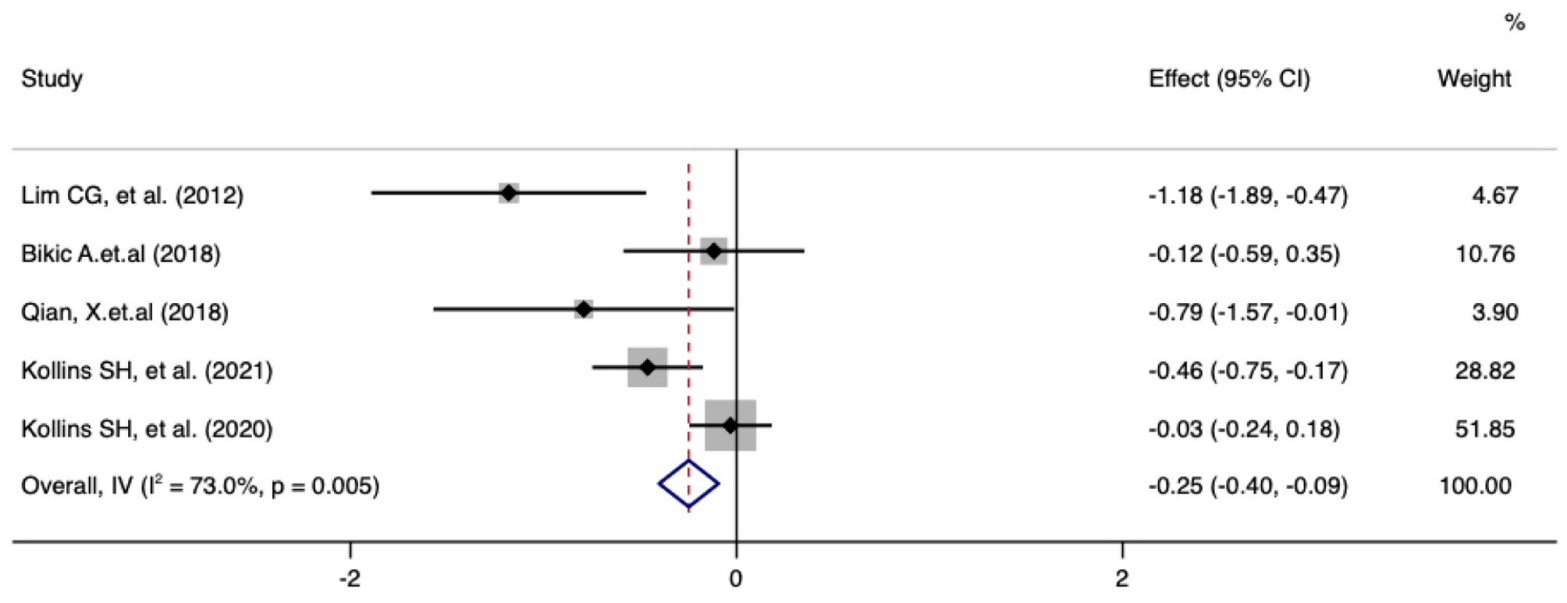
Forest plot of treatment effect of digital therapeutics on ADHD-RS Inattention score in five studies.

Meta-analysis of the effect of digital therapeutics on the inattention score of the ADHD-RS showed that the combined effect from five studies was −0.25 (−0.40, −0.09) with *P* = 0.002. This result suggests that digital interventions significantly improved attention in pediatric ADHD patients. To further explore the heterogeneity among included studies, chi-square and I^2^ analyses were performed, and small differences in heterogeneity were observed between treatment groups (I^2^ = 73.0%, *P* = 0.005) (Figure 2).

### Hyperactivity improvement based on ADHD-RS score after treatment with digital therapeutics

**Figure 3:**
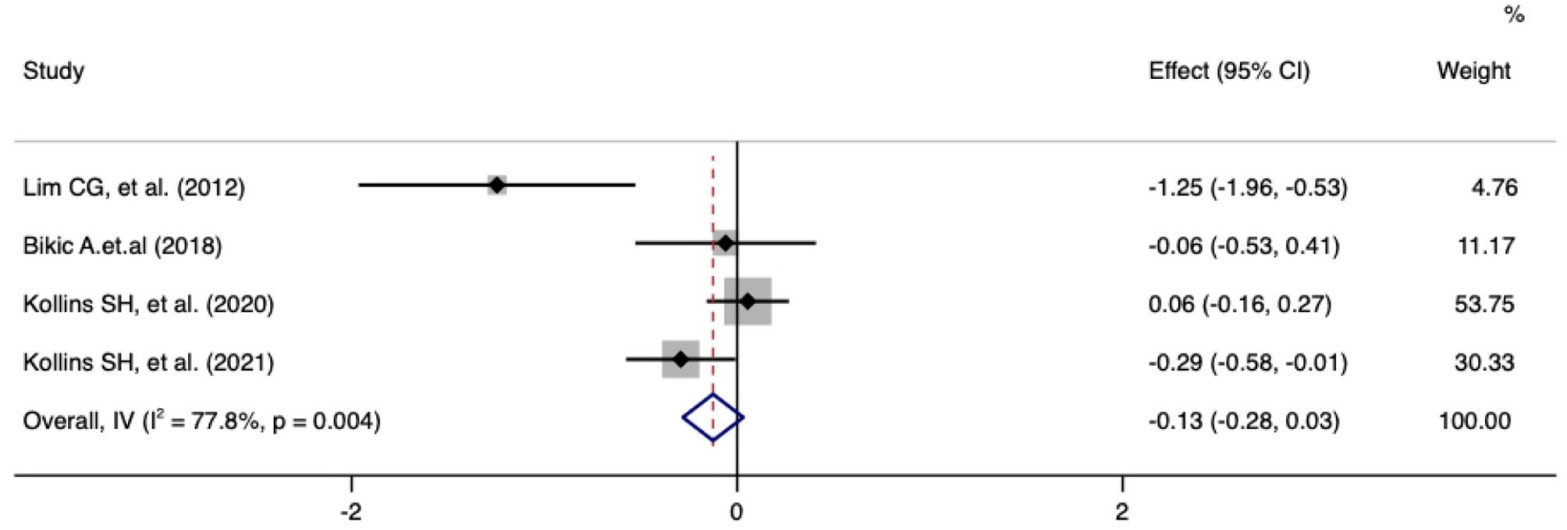
Forest plot of treatment effect of digital therapeutics on ADHD-RS hyperactivity score in five studies.

Meta-analysis of the effect of digital therapeutics on the ADHD-RS hyperactivity score showed that the combined effect from five studies was −0.13 (−0.28, 0.03) with *P* = 0.018. This result suggests that digital interventions improved hyperactivity symptoms in pediatric ADHD patients. Chi-square and I-square analyses revealed differences in heterogeneity between treatment groups (I^2^ = 77.8%, *P* = 0.004) (Figure 3).

### General improvement of ADHD-RS score after treatment with digital therapeutics

**Figure 4:**
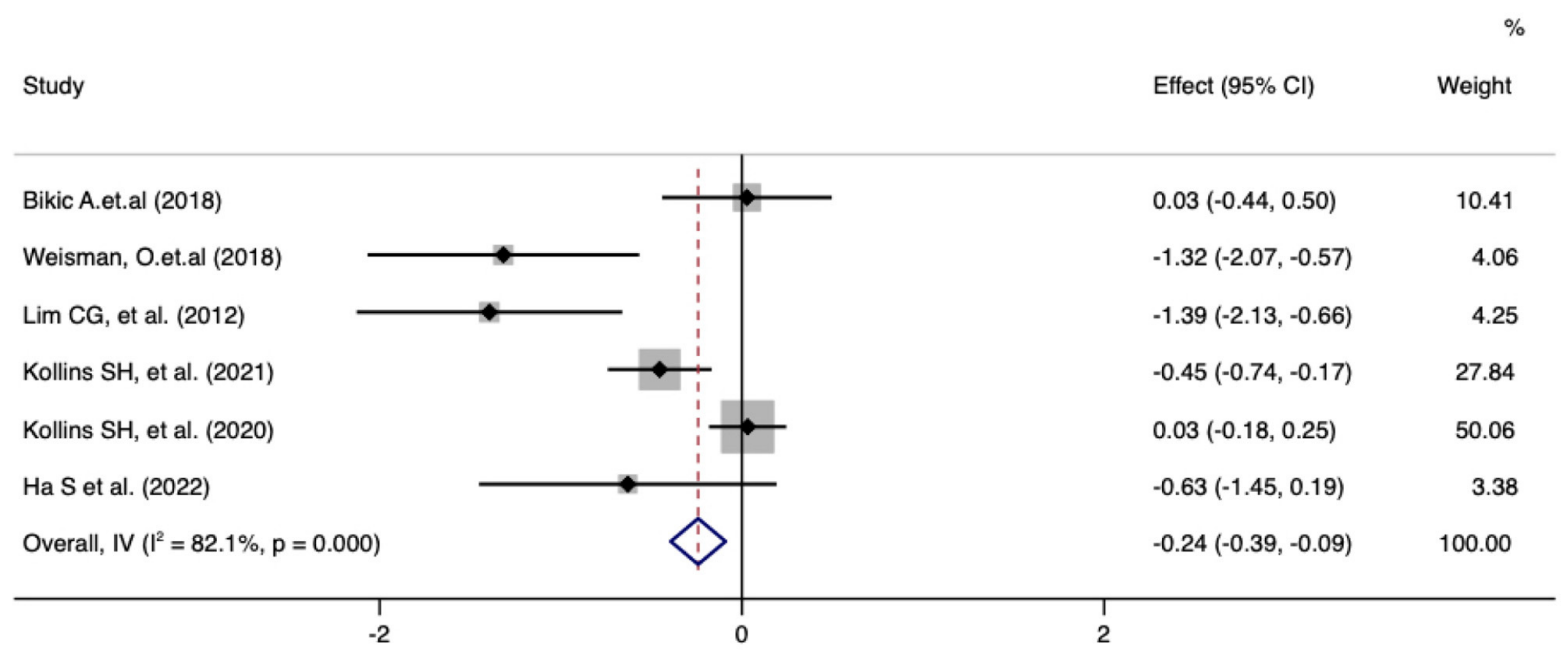
Forest plot of the treatment effect of digital therapeutics on the total ADHD-RS score in six studies.

Meta-analysis of the effect of digital therapeutics on the total ADHD-RS score showed that the combined effect from six studies was −0.24 (−0.39, −0.09) with *P* = 0.013. This result suggests that digital interventions led to significant improvement in the total ADHD-RS score for pediatric ADHD patients. Chi-square and I-square analyses identified significant differences in heterogeneity between treatment groups (I^2^ = 82.1%, *P* = 0.000) (Figure 4).

### Publication bias and sensitivity analysis of studies reporting ADHD-RS scores

**Figure 5:**
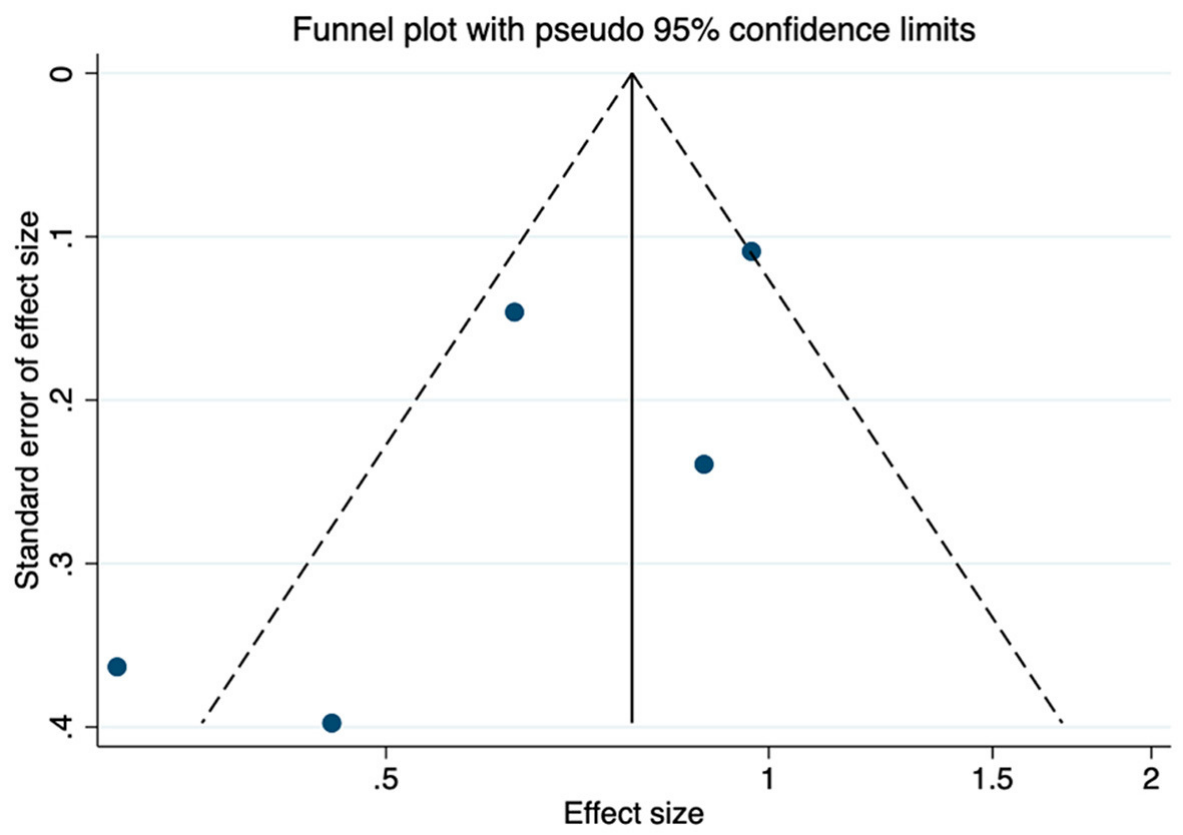
Funnel plot showing publication bias among studies reporting ADHD-RS scores.

**Figure 6:**
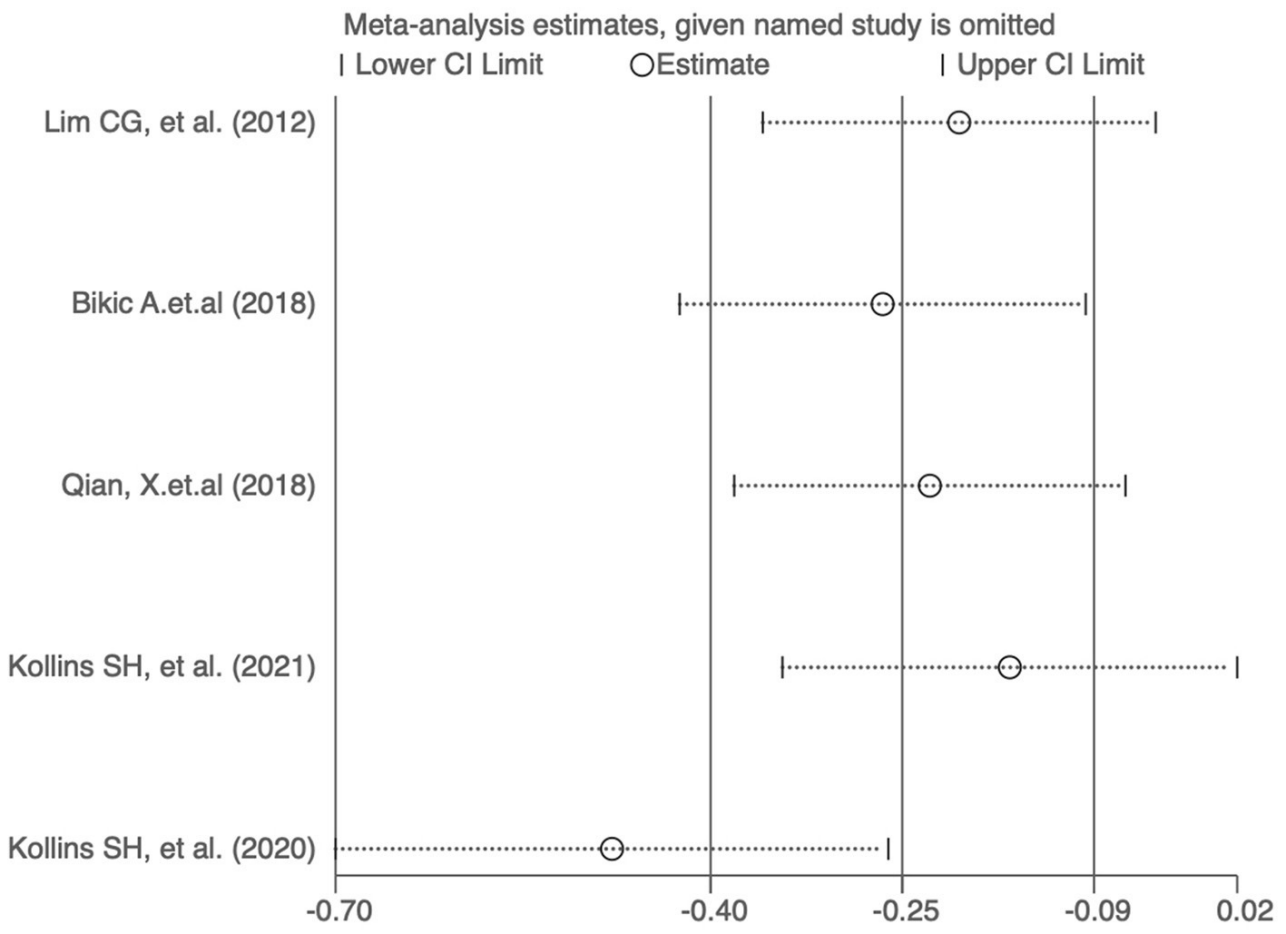
Sensitivity assessment of the cumulative meta-analysis results regarding the effects of digital therapeutics on ADHD-RS scores.

Funnel plots were used to assess the publication bias of the included studies reporting ADHD-RS scores in the meta-analysis, and quantitative assessment was performed using Egger's test. As shown in Figure 5, no obvious publication bias was observed (*P* = 0.707). We next investigated the influence of each individual study on the summary assessment of the overall meta-analysis. The recalculated ORs with the exclusion of each study were not significantly altered (Figure 6). Therefore, the results of the analyses involving ADHD-RS scores are believed to be statistically reliable, and individual studies had only a minor effect on the pooled estimations.

### Decreased reaction time in CPTs after treatment with digital therapeutics

Compared with most ADHD assessment tools relying on subjective judgment, CPTs are designed to provide objective, reliable information for the assessment of inattention symptoms. Although neuropsychological tasks can vary among different CPTs, the reaction time in CPT tasks is directly related to ADHD inattention severity. In our analysis, reaction time in CPT tasks was normalized in different studies, and the effects of digital therapeutics on CPT and inattention symptoms were examined.

**Figure 7:**
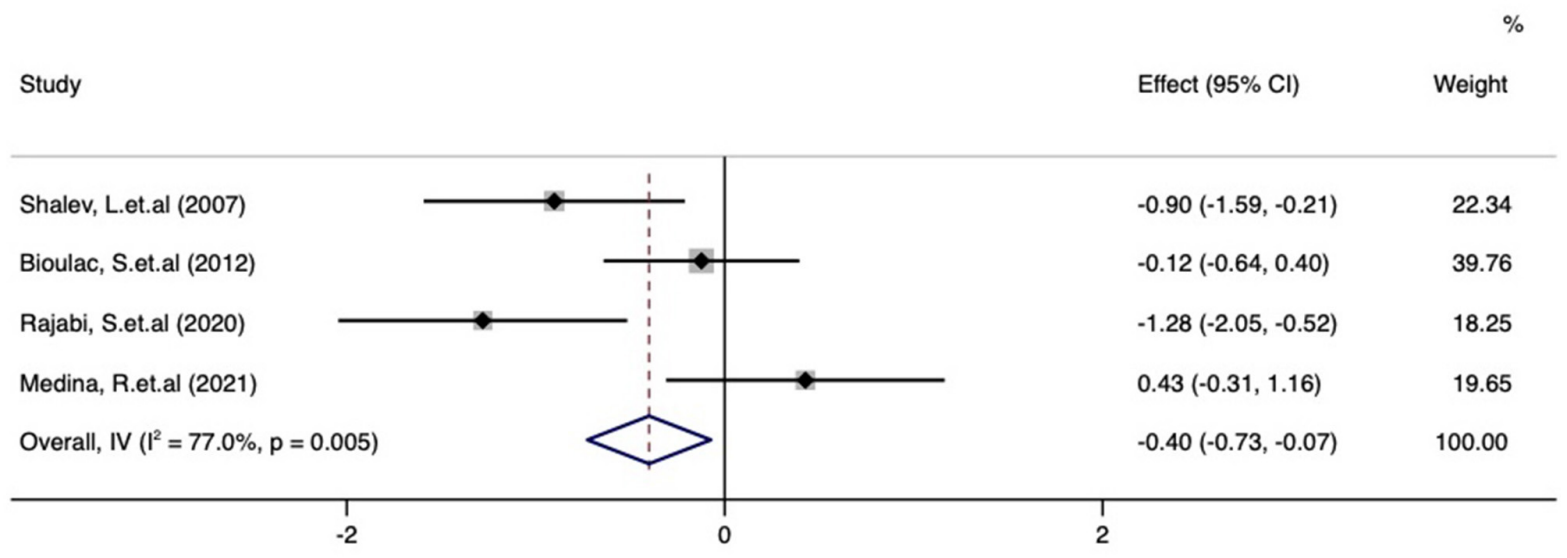
Forest plot of the treatment effect of digital therapeutics on reaction time in CPTs in four studies.

Meta-analysis of the effect of digital therapeutics on reaction time in CPTs among pediatric ADHD patients, the combined effect from the four studies was −0.40 (−0.73, −0.07) with *P* = 0.016. This result suggests that digital interventions significantly decreased the reaction time in CPT with the improvement of inattention symptoms in ADHD patients. Chi-square and I-square analyses to explore the heterogeneity among the included studies revealed differences in heterogeneity between treatment groups (I^2^ = 77.3%, *P* = 0.004) (Figure 7).

### Publication bias and sensitivity analysis of studies reporting CPT results

**Figure 8:**
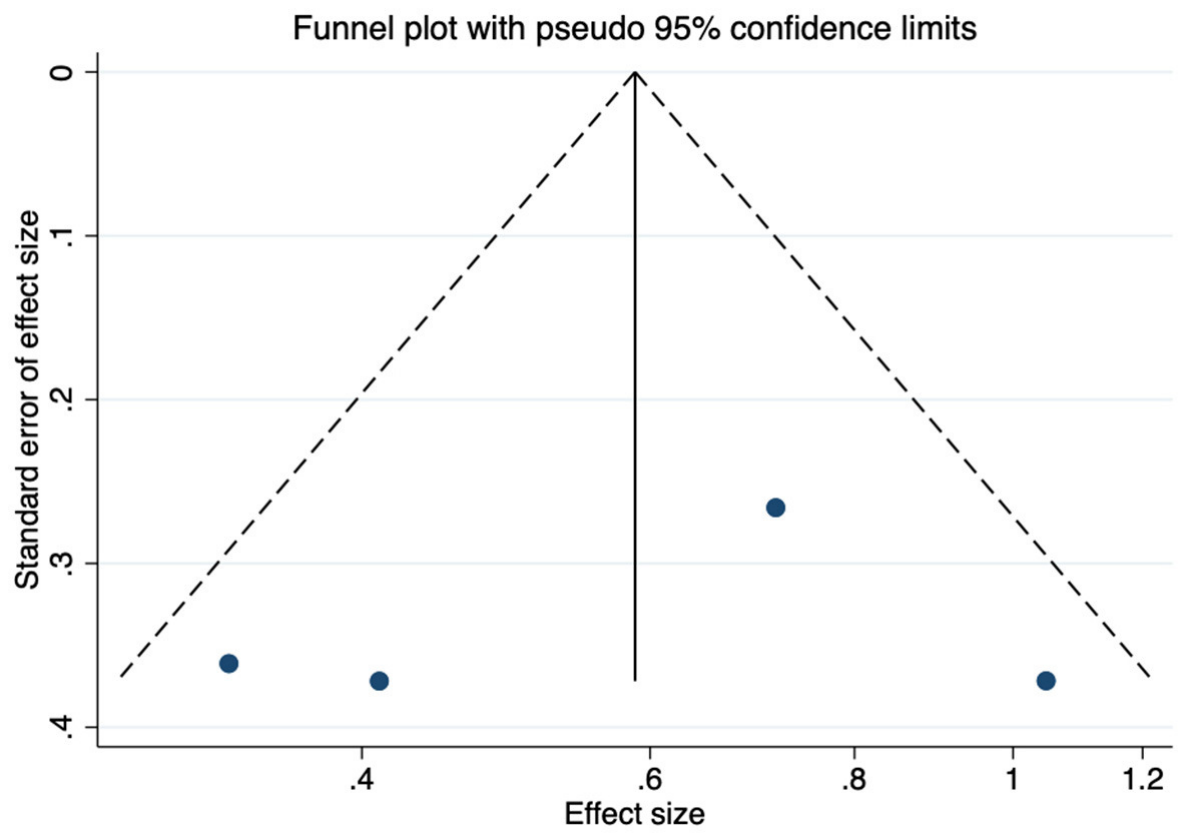
Funnel plot showing publication bias among studies reporting CPT results.

**Figure 9:**
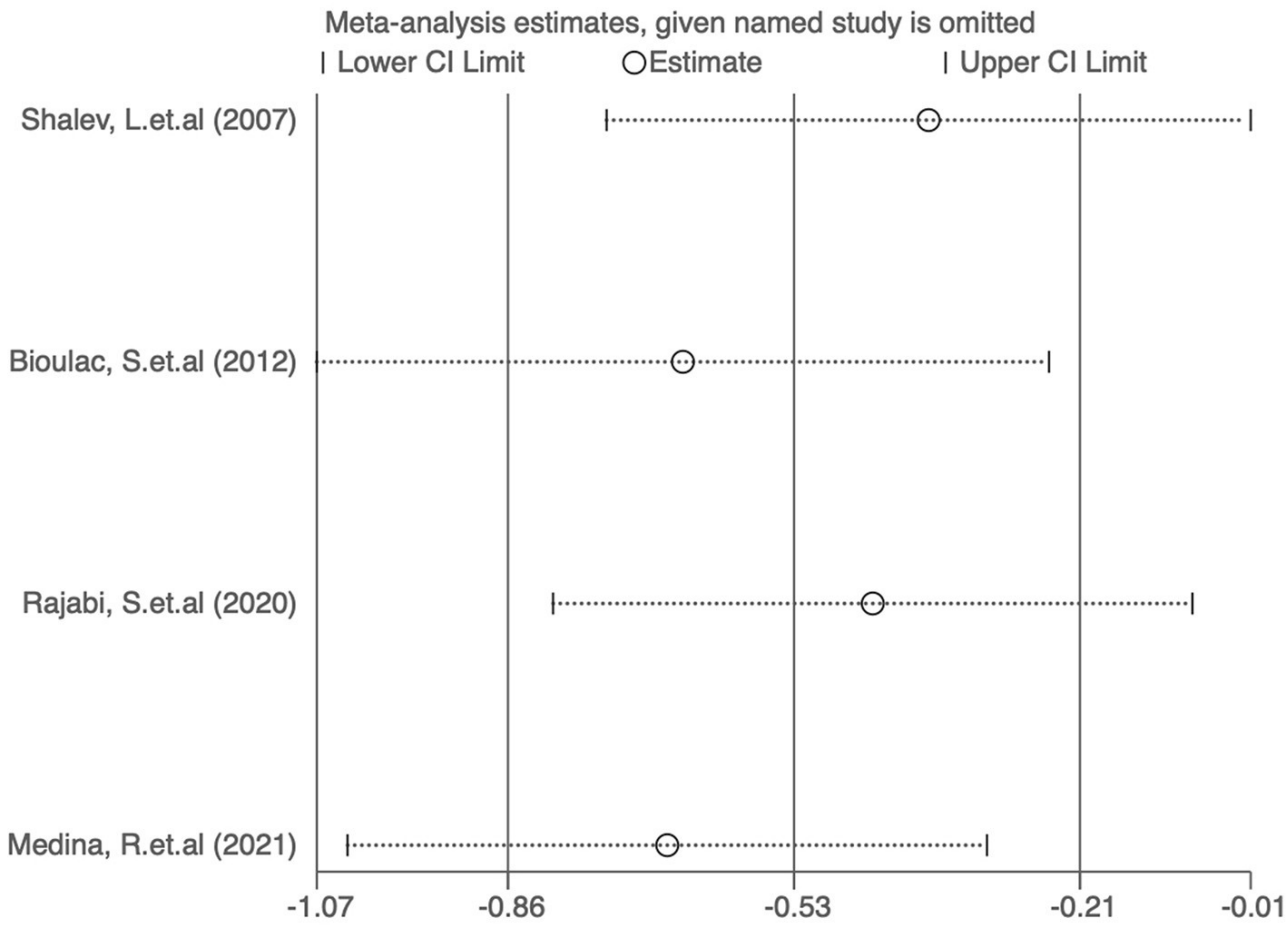
Sensitivity analysis of the cumulative meta-analysis results regarding the effects of digital therapeutics on CPT results.

As shown in Figure 8, no obvious publication bias was observed among the studies reporting CPT results (*P* = 0.83). We next investigated the influence of each individual study on the summary assessment from the overall meta-analysis (Figure 9). The recalculated ORs were not significantly altered upon removal of each study individually. Therefore, the results of the analyses of reaction time in CPTs are believed to be statistically reliable, and individual studies had only a minor effect on the pooled estimations.

### Executive functions after treatment with digital therapeutics

**Figure 10:**
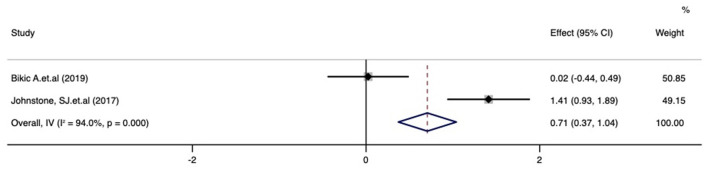
Forest plot of the treatment effect of digital therapeutics on the BRIEF executive function score in four studies.

Meta-analysis of the effect of digital therapeutics on BRIEF executive function score showed that the combined effect from two studies was 0.71 (0.37, 1.04) with *P* = 0.082. This result suggests that digital interventions significantly improved executive functions in pediatric ADHD patients. Chi-square and I-square analyses revealed differences in heterogeneity between treatment groups (I^2^ = 94.0%, *P* = 0.000) (Figure 10).

### Working memory improvement after treatment with digital therapeutics

**Figure 11:**
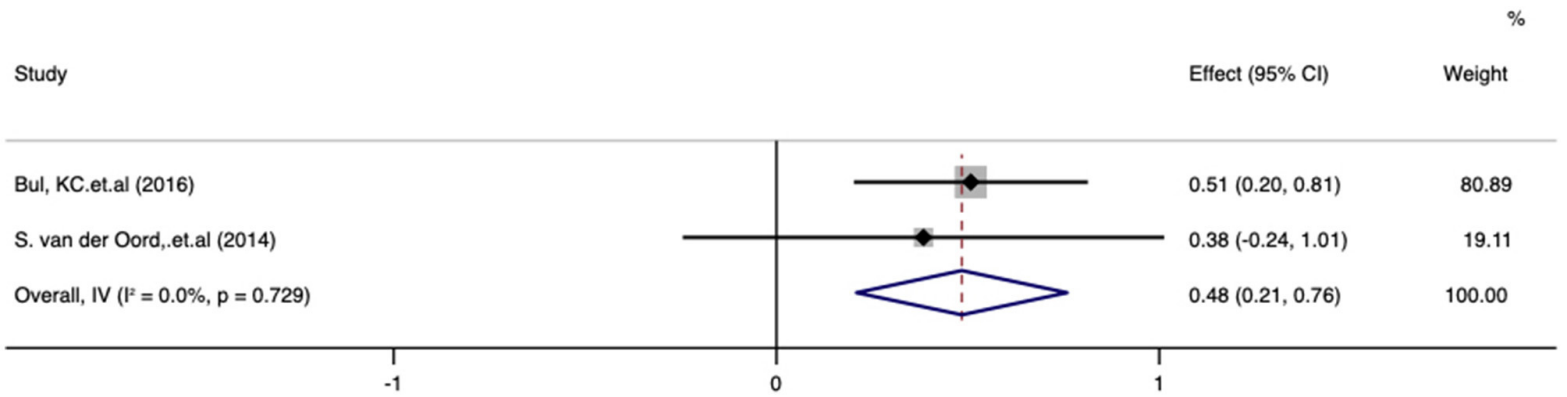
Forest plot of the treatment effect of digital therapeutics on the BRIEF working memory score in three studies.

Meta-analysis of the effect of digital therapeutics on BRIEF working memory score showed that the combined effect from three studies was 0.48 (0.21, 0.76) with *P* = 0.001. This result suggests that digital interventions significantly improved working memory in pediatric ADHD patients. Chi-square and I-square analyses detected no differences in heterogeneity between treatment groups (I^2^ = 0%, *P* = 0.729), but heterogeneity was not observed within the three included studies (Figure 11).

### Publication bias and sensitivity analysis of studies reporting BRIEF scores

**Figure 12:**
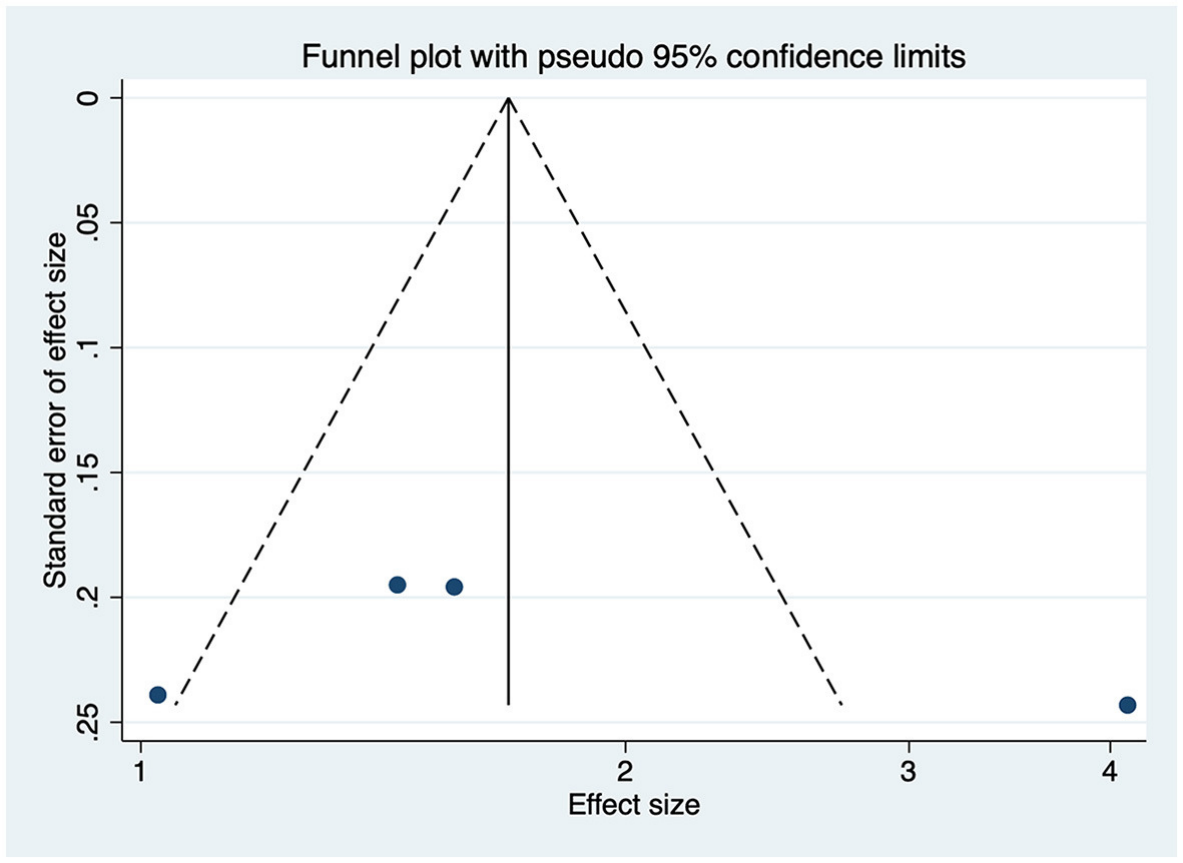
Funnel plot showing publication bias among studies reporting BRIEF scores.

**Figure 13:**
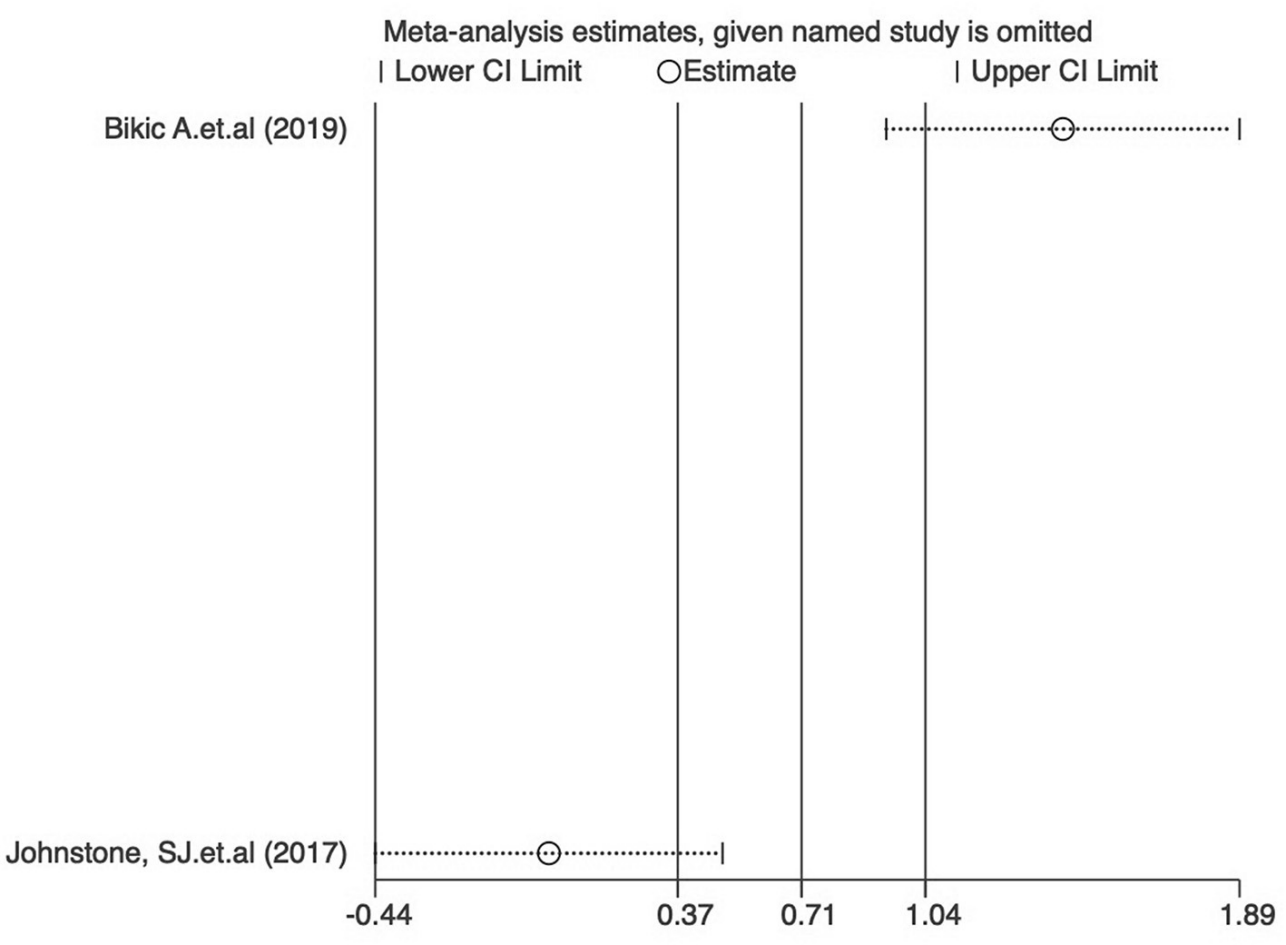
Sensitivity analysis of the cumulative meta-analysis results regarding the effects of digital therapeutics on BRIEF scores.

As shown in Figure 12, no obvious publication bias was observed among studies reporting BRIEF scores (*P* > 0.05). We next investigated the influence of each individual study on the summary assessment of the overall meta-analysis (Figure 13). The recalculated ORs were not significantly altered upon the removal of each study individually. Therefore, the present results are believed to be statistically reliable, and individual studies had only a minor effect on the pooled estimations.

### Effect of personalization on the efficacy of digital therapeutics

**Figure 14:**
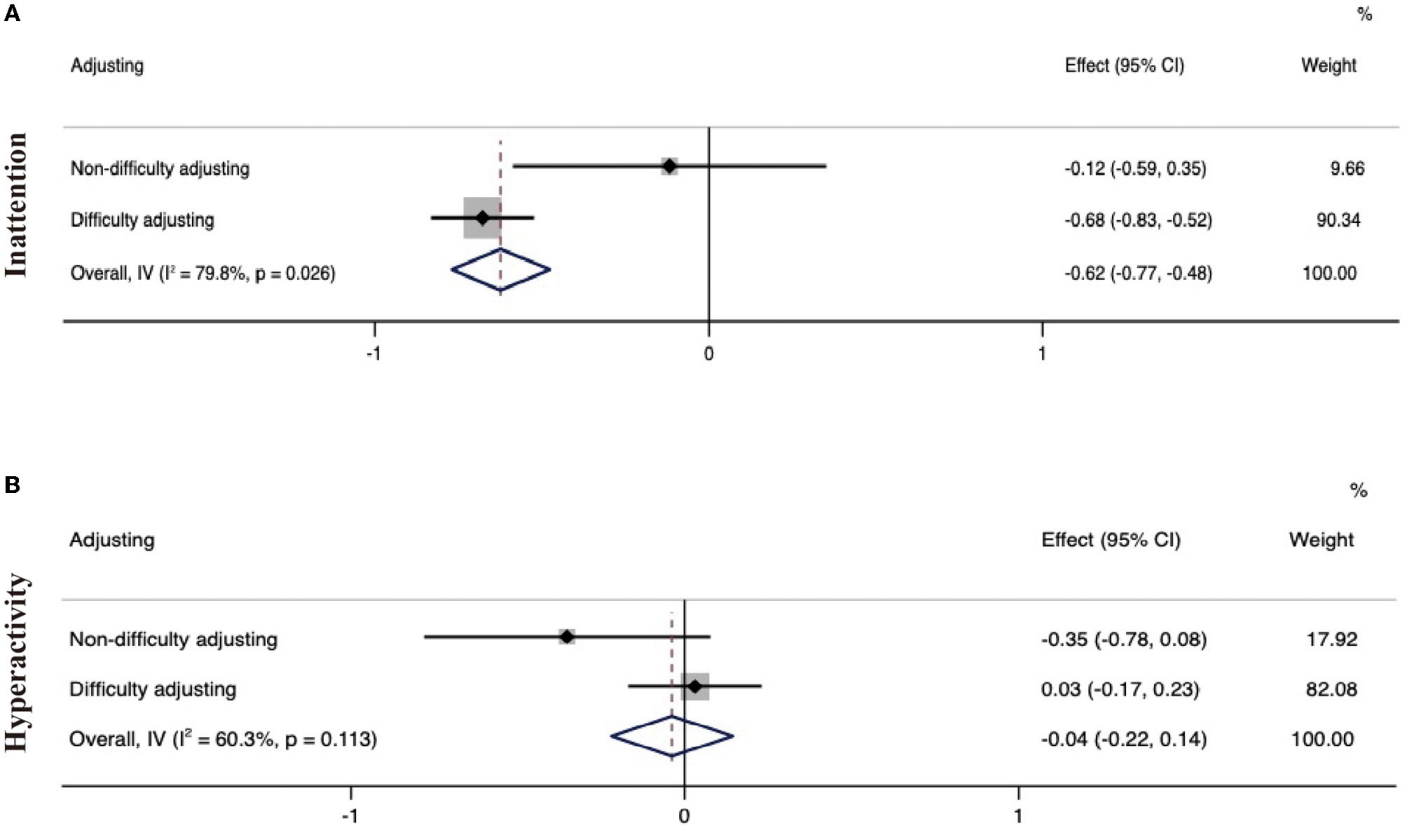
Forest plot of the treatment effect of digital therapeutics according to the presence of personalized difficulty adjustment. **(A)** Forest plot of treatment effect on inattention; **(B)** Forest plot of treatment effect on hyperactivity.

Subgroup analysis according to the characteristics of digital therapy showed that digital therapy with personalized difficulty adjustment had a better therapeutic effect on inattention (−0.68 [−0.83, −0.52]) than digital therapeutics without a difficulty-adjusting system (−0.12 [−0.59, 0.35]). However, digital therapy without personalized difficulty adjustment had a more pronounced positive effect on ADHD-related hyperactivity (−0.35 [−0.78, 0.08] vs. 0.03 [−0.17, 0.23], Figure 14).

### Efficacy of digital therapeutics in different regions

To further explore the different efficacy of digital therapeutics in different regions, the results of subgroup analysis revealed that Asian ADHD patients achieved a better therapeutic effect on inattention relative to patients in Europe (−0.49 [−0.97, 0.00] vs. −0.14 [−0.45, 0.18]). Similarly, on executive functions, Asian ADHD patients receiving digital therapeutics showed a better performs compared with that in Europe (7.58 [6.61, 8.55] vs. −0.32 [−0.52, −0.12], Supplementary Figure 1).

## Discussion

The present meta-analysis of 31 studies investigated the overall effectiveness of digital therapeutics for pediatric ADHD from the perspective of ADHD core symptom changes, ADHD cognitive functioning based on working memory and executive functioning, and reaction time in attention performance derived from CPTs completed by the patients themselves. The effect size for inattention symptom alleviation was −0.25 (−0.40, −0.09), and a decrease in CPT reaction time also was observed (effect −0.40 [−0.73, −0.07]), revealing a positive effect of digital therapeutics on inattention symptoms and functioning. Measures of executive function (effect 0.71 [0.37, 1.04]) also were improved based on data from two studies. The score for impulsive hyperactivity was slightly decreased after treatment (effect −0.13 [−0.28, 0.03]). Additionally, working memory appeared to be increased, but the different between the treated and control groups was not significant. Visual appraisal of the sensitivity analysis results suggested the absence of publication bias. Together these results indicate that digital interventions could be a possible therapeutic strategy for pediatric ADHD, especially with regard to attention control.

Previous studies of the use of digital therapeutics for ADHD treatment have provided preliminary evidence of their effectiveness. Multiple studies showed that video games can improve cognition by promoting the formation and restructuring of neurobiological pathways (51, 52), and other studies have provided support for the clinical benefit of digital therapeutics for depression and anxiety in ADHD (53, 54). Furthermore, previous studies have consistently shown that digital therapeutics can improve the social skills of ADHD patients (55, 56). Previous reviews have also explored the potential of video games for use in child healthcare, and their effects during the intervention phase were reported to be positive (55, 56). Thus, digital therapy is accepted as a useful strategy to improve patients' social and neurocognitive skills related to certain interventions and could contribute to the assessment and management of ADHD.

Digital therapeutics was defined by the Digital Therapeutics (DTx) Alliance as “delivering evidence-based therapeutic interventions to patients that are driven by software to prevent, manage, or treat a medical disorder or disease.” To date, a broad spectrum of digital therapeutics has been recommended and adopted to treat developmental, behavioral, and emotional disorders in children. However, these digital treatments are based on different treatment mechanisms, which is referred to as the “active component”, and supported by various forms of technology, including big data, artificial intelligence, sensor technology, video-game and virtual reality. Because of the heterogeneous nature of these digital treatments, several issues remain to be addressed, including: (1) the lack of consensus about the outcome measures for efficacy; (2) the limited validation of the digital devices in non-English languages; (3) the lack of digital treatment categorization based on the “active component” and technology platform; and (4) the lack of standardized protocols for intervention based on categorization. Thus, there is an urgent need for methodologically robust, adequately powered research evaluating the safety, efficacy, and effectiveness of digital interventions for children and young people with ADHD, especially digital therapeutics in languages other than English. This research may require the inclusion of children and adolescents as well as therapists in the product design and development processes to ensure the interventions are fit for purpose and user-centered. Continuous evaluation of evolving interventions is also necessary.

Another important issue in digital therapy studies is the choice of the control group. Although recent clinical trials have applied different type of digital interventions as control groups, waitlist studies were more common. There is a need to further clarify the potential adverse effects of digital therapy. Video game therapy was reported to lead to symptoms of headache, dizziness, agitation, and other adverse effects (56). Moreover, patients with ADHD are likely to be at higher risk for video game addiction. With deficits in behavioral response inhibition and self-control, children with ADHD may be unable to resist inner and environmental interference and be prone to develop an internet addiction. Therefore, for administration of video game therapy, the time and frequency of video game play should be strictly planned.

One method for categorizing digital therapeutics is by their core active component, which is considered reflective of the “therapeutic mechanisms” of digital interventions. Mechanisms of digital therapies include neuroplasticity and neural reorganization boosting through cognitive training. Cognitive training includes a series of tasks designed to improve one or more aspects of executive function, such as attention, working memory, reaction time, cognitive flexibility, and motor performance (57). In addition to training related cognitive functions, Video game therapy also introduces new experience gained from previous training (51). Further subgroup analyses of patients receiving digital therapy are needed to determine whether any core components and technology platforms are superior. Such studies could address whether an artificial intelligence (AI) engine that empowers personalized difficulty adjustment will achieve higher efficacy. The present study could not provide a clear answer to such questions due to limited sample sizes and the lack of product descriptions in the included studies.

A major limitation of the implementation of digital therapy in clinical practice is the ongoing need to identify the best intervention protocols, efficacy, safety, feasibility, and benefit-to-cost ratio of these therapies. Currently, clinicians should be cautious about recommending different digital therapies, because even those that appear similar may have different core mechanisms and technology platforms and thus offer differing efficacies.

A strength of the present study was the use of reliable quantitative evaluation methods to systematically compare the clinical efficacy of digital therapy in pediatric ADHD. Based on the current evidence in the literature, we explored important elements of interventions that can be specifically linked to their effects on outcomes including inattention, hyperactivity, executive function, and working memory. In previous studies, essential elements of behavior or digital interventions were frequently omitted in the data synthesis and interpretation. This led to the duplication of effort, uncertainty, and confusion and undermined the potential to accumulate evidence across studies for determining the efficacy of specific therapeutic approaches. This also points to the urgent need for the identification and analysis of essential elements in digital treatment (58, 59).

The present study also has several limitations. Firstly, the scales adopted for ADHD symptom assessment were inconsistent, which limited the interpretation of treatment outcomes. More studies employing the same assessment scales are needed in the future to obtain more reliable results (60). Secondly, the small number of included studies also limits the generalizability of the results of this meta-analysis. The study conclusions may have been affected by sample size and the risk of probable bias. In our study, single centered and open-labeled studies were included and may not be representative of larger populations which could affect the generalizability of our findings. Despite these limitations, we believe that our study provides important insights into the efficacy of digital intervention, and we hope that it will stimulate further research in this area.

In summary, the results of our study suggest that digital therapy, mostly in the form of video game-based technology, offers potential clinical efficacy for improving attention, sensory perception, and a variety of cognitive functions in pediatric ADHD patients. However, additional research is needed to identify the optimal conditions for digital therapy as well as any potential adverse effects (e.g., persistence of efficacy, optimal duration of treatment, addiction problems, as well as clinical applications). Overall, based on the existing literature evidence, we conclude that digital interventions demonstrate possible efficacy on improving clinical symptoms of pediatric ADHD.

## Data availability statement

The original contributions presented in the study are included in the article/Supplementary material, further inquiries can be directed to the corresponding author.

## Author contributions

All authors listed have made a substantial, direct, and intellectual contribution to the work and approved it for publication.

## Supplementary material

Supplementary Figure 1 Efficacy of digital therapeutics in different regions. **(A)** Forest plot of treatment effect of digital therapeutics on inattention in Asia and Europe; **(B)** Forest plot of the treatment effect of digital therapeutics on executive functions in Asia and Europe.
