# Supplementary figures and images for: Meta-analysis of the efficacy of digital therapies in children with attention-deficit hyperactivity disorder
Source: Front Psychiatry. 2023 May 16;14:1054831. doi: 10.3389/fpsyt.2023.1054831 (PMC10228751; doi:10.3389/fpsyt.2023.1054831)

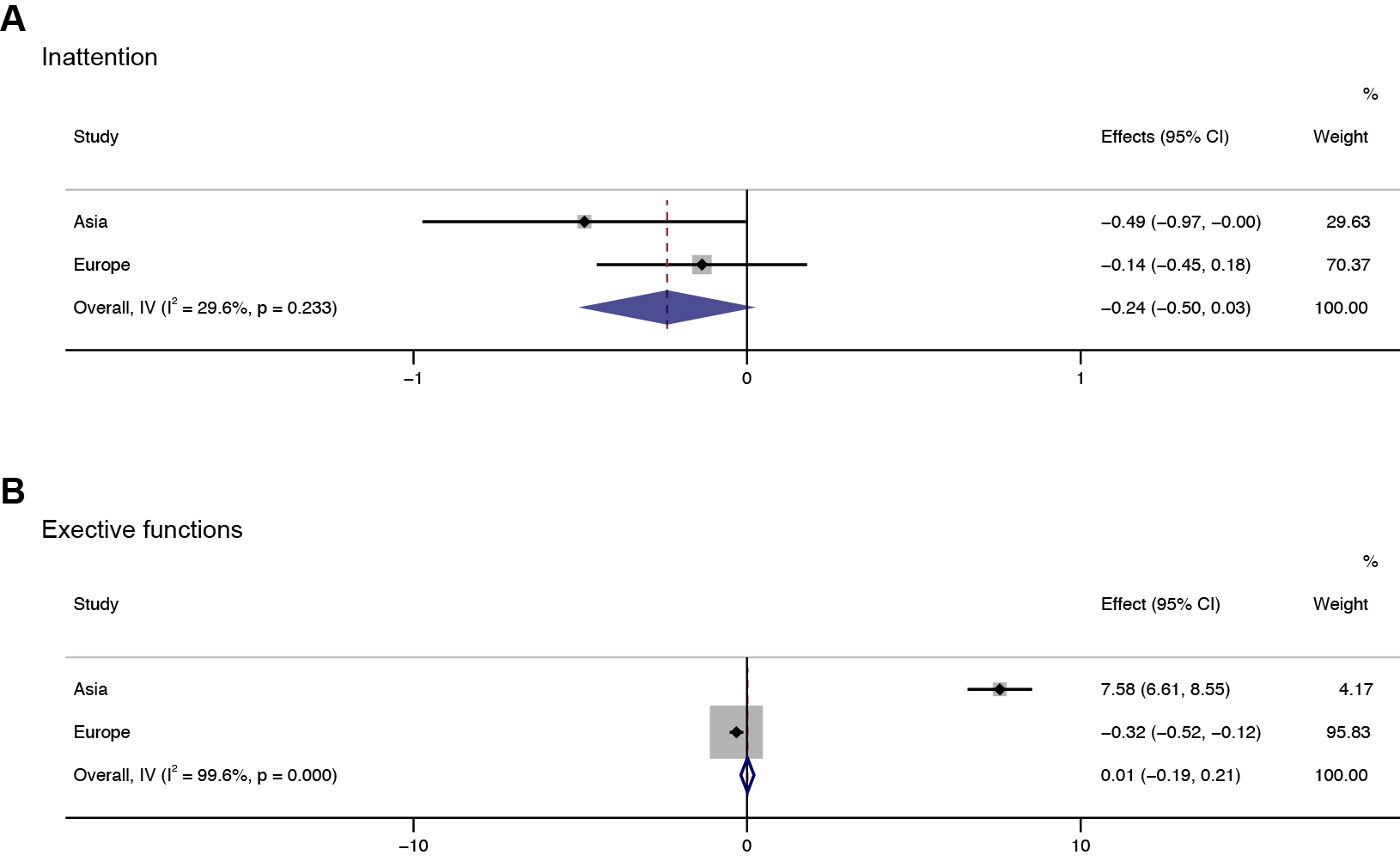

Supplement: Supplementary Figure 1 — Efficacy of digital therapeutics in different regions. (A) Forest plot of treatment effect of digital therapeutics on inattention in Asia and Europe; (B) Forest plot of the treatment effect of digital therapeutics on executive functions in Asia and Europe. [file Image_1.TIF]
